# Genome-wide association study of copy number variation with lung function identifies a novel signal of association near *BANP* for forced vital capacity

**DOI:** 10.1186/s12863-016-0423-0

**Published:** 2016-08-11

**Authors:** Nick Shrine, Martin D. Tobin, Claudia Schurmann, María Soler Artigas, Jennie Hui, Terho Lehtimäki, Olli T. Raitakari, Craig E. Pennell, Qi Wei Ang, David P. Strachan, Georg Homuth, Sven Gläser, Stephan B. Felix, David M. Evans, John Henderson, Raquel Granell, Lyle J. Palmer, Jennifer Huffman, Caroline Hayward, Generation Scotland, Anders Malarstig, Bill Musk, Alan L. James, UK BiLEVE, Louise V. Wain

**Affiliations:** 1 Department of Health Sciences, University of Leicester, University Road, Leicester, LE1 7RH UK; 2 National Institute for Health Research (NIHR) Leicester Respiratory Biomedical Research Unit, Glenfield Hospital, Leicester, LE3 9QP UK; 3 Department of Functional Genomics, University Medicine Greifswald, Interfaculty Institute for Genetics and Functional Genomics, Greifswald, Germany; 4 DZHK (German Center for Cardiovascular Research), partner site Greifswald, 17475 Greifswald, Germany; 5 Busselton Population Medical Research Institute, Sir Charles Gairdner Hospital, Nedlands, Australia; 6 Department of Clinical Chemistry, Fimlab Laboratories, University of Tampere and Tampere University Hospital, Tampere, 33521 Finland; 7 Department of Clinical Physiology, Research Centre of Applied and Preventive Cardiovascular Medicine, University of Turku, Turku University Hospital, Turku, 20521 Finland; 8 School of Women’s and Infants’ Health, The University of Western Australia, Perth, Australia; 9 Division of Population Health Sciences, St. George’s University of London, London, UK; 10 Department of Internal Medicine B – Pulmonary Medicine, Weaning and Infectious Diseases and Scientific Division of Pneumology and Pneumological Epidemiology, University Medicine Greifswald, Greifswald, Germany; 11 Department of Internal Medicine B – Cardiology, University Medicine Greifswald, Greifswald, Germany; 12 MRC Integrative Epidemiology Unit, University of Bristol, Bristol, UK; 13 School of Social and Community Medicine, University of Bristol, Bristol, UK; 14 University of Queensland Diamantina Institute, Translational Research Institute, Brisbane, Queensland Australia; 15 School of Public Health, University of Adelaide, Adelaide, Australia; 16 Institute of Genetics and Molecular Medicine, University of Edinburgh Western General Hospital, Crewe Road, Edinburgh, EH4 2XU UK; 17 Pfizer Worldwide Research and Development, Sollentuna, Sweden; 18 Department of Respiratory Medicine, Sir Charles Gairdner Hospital, Nedlands, Australia; 19 Department of Pulmonary Physiology and Sleep Medicine, Sir Charles Gairdner Hospital, Nedlands, Australia

**Keywords:** Copy number variation, Lung function, Genome-wide association study

## Abstract

**Background:**

Genome-wide association studies of Single Nucleotide Polymorphisms (SNPs) have identified 55 SNPs associated with lung function. However, little is known about the effect of copy number variants (CNVs) on lung function, although CNVs represent a significant proportion of human genetic polymorphism. To assess the effect of CNVs on lung function quantitative traits, we measured copy number at 2788 previously characterised, common copy number variable regions in 6 independent cohorts (*n* = 24,237) using intensity data from SNP genotyping experiments. We developed a pipeline for genome-wide association analysis and meta-analysis of CNV genotypes measured across multiple studies using SNP genotype array intensity data from different platform technologies. We then undertook cohort-level genome-wide association studies of CNV with lung function in a subset of 4 cohorts (*n* < =12,403) with lung function measurements and meta-analysed the results. Follow-up was undertaken for CNVs which were well tagged by SNPs, in up to 146,871 individuals.

**Results:**

We generated robust copy number calls for 1962 out of 2788 (70 %) known CNV regions genome-wide, with 1103 measured with compatible class frequencies in at least 2 cohorts. We report a novel CNV association (discovery *P* = 0.0007) with Forced Vital Capacity (FVC) downstream of *BANP* on chromosome 16 that shows evidence of replication by a tag SNP in two independent studies (replication *P* = 0.004). In addition, we provide suggestive evidence (discovery *P* = 0.0002) for a role of complex copy number variation at a previously reported lung function locus, containing the rootletin gene *CROCC,* that is not tagged by SNPs*.*

**Conclusions:**

We demonstrate how common CNV regions can be reliably and consistently called across cohorts, using an existing calling algorithm and rigorous quality control steps, using SNP genotyping array intensity data. Although many common biallelic CNV regions were well-tagged by common SNPs, we also identified associations with untagged mulitallelic CNV regions thereby illustrating the potential of our approach to identify some of the missing heritability of complex traits.

**Electronic supplementary material:**

The online version of this article (doi:10.1186/s12863-016-0423-0) contains supplementary material, which is available to authorized users.

## Background

Genome-wide association studies (GWAS) of single nucleotide polymorphisms (SNPs) have highlighted genes and biological pathways associated with risk of a variety of diseases and variability in quantitative health-related traits. Quantitative lung function traits are of major public health relevance and large GWAS have identified multiple common variants which collectively explain only a small proportion of the phenotypic variance. For lung function traits (forced expired volume in 1 second, FEV_1_, forced vital capacity, FVC and the ratio FEV_1_/FVC), the 55 SNPs reported in recent large GWAS explain 6.6 % of the variance in FEV_1_/FVC, 5.3 % of the variance in FEV_1_ and 4.0 % for FVC [1–6].

Copy number variants (CNVs) are deletions or duplications of parts of a chromosome, ranging from a few hundred to a few million base pairs in length [7], leading to extra or fewer copies of a certain DNA sequence relative to the usual 2 copies for a diploid genome. CNVs have been associated with several disorders including Crohn’s disease, rheumatoid arthritis and diabetes [8, 9] and neurological disorders such as schizophrenia [10, 11], autism [12, 13] and developmental delay [14], although contribution of CNVs to phenotypic variance is still uncertain for many common traits [9, 15, 16]. To date, no genome-wide survey of the effect(s) of copy-number variants on lung function quantitative traits has been carried out.

Platforms primarily designed for SNP genotyping can additionally provide an estimate of copy number by looking for deviations in the intensity signal from each allelic SNP probe; “non-polymorphic” probes (probes at genomic positions where there is no known SNP) have been added to some GWAS platforms specifically for this purpose. The wide availability of these intensity data presents an opportunity to measure genome-wide copy number variation across multiple studies and bring together large sample sizes for association testing.

Here we describe a pipeline for genome-wide CNV genotype measurement using SNP array intensity data. We first tested the pipeline and defined appropriate quality control filters using 6 cohorts comprising intensity data from 5 different genotyping platforms. We then undertook an analysis of genome-wide CNV associations with lung function for up to 12,403 individuals in a subset of 4 cohorts with lung function measurements at 1962 copy number variable loci. We show how this approach can identify multiallelic variants that are not well-tagged by SNPs and that potentially explain some of the missing heritability of lung function quantitative traits.

## Methods

There were 2 stages of the analysis. In the first stage we refined a pipeline for copy number calling to determine the quality control steps required to obtain consistent copy number measurement across multiple cohorts and genotyping platforms. In this stage we analysed intensity data from SNP genotyping platform experiments for 24,237 samples from 6 different cohorts (Busselton Health Study (BHS, *n* = 3496), Young Finns Study (YFS, *n* = 2682), British 1958 Birth Cohort (B58C, *n* = 2920), Study of Health in Pomerania (SHIP, *n* = 4072), Raine Study (*n* = 1685) and the Avon Longitudinal Study of Parents and Children (ALSPAC, *n* = 9382)). The aim of this first stage was to identify whether common CNVs could be measured with consistency across multiple cohorts with different intensity data. In the second stage we tested for association of copy number with lung function in 4 cohorts which had lung function measurements. Lung function association results were then meta-analysed across cohorts.

In stage 1 we selected known, common copy number variable regions (CNVRs) from a map of 11,700 copy number variants across the genome published by The Genome Structural Variation Consortium [17], which included 3276 autosomal CNVRs observed twice or more in 20 HapMap CEU samples. Of these, 2788 CNVRs were retained after quality control (Additional file 1).

The Log R Ratio (LRR) of the intensity signals from the allelic probes used in the SNP genotyping was used to estimate copy number. LRR is the ratio of the intensity to the expected intensity of the relevant genotype cluster and is a transformation of the raw X and Y intensity signals that enhances the correlation with copy number. The LRR was available for all of the cohorts apart from ALSPAC where the sum of the X and Y intensities from each of the allelic probes was used instead. Replicate samples and samples which failed intensity noise filtering (Additional file 1) were excluded before copy number calling. Where SNP genotypes were available, we derived ancestry-informative principal components using EIGENSOFT [18] and removed any samples that had a score on any of the first 4 principal components greater than 6 standard deviations from the mean.

At each CNVR the samples passing the above quality-control filters were clustered by a 1-dimensional summary of the intensity signals from the probes within the CNVR boundary; only samples with non-missing data for these probes were included when clustering each CNVR. The cluster to which a sample belongs was used as a proxy for the copy number class of the sample at that CNVR. The clustering was performed by Bayesian hierarchical mixture modelling implemented in the R package CNVCALL [9, 19].

For the initial run of clustering no prior information about the number of classes was included, with the algorithm determining the most likely number of classes from the data independently for each cohort. In the absence of a “gold standard” for the correct number of classes at each CNVR, we used the number of classes called by the best-performing cohort as our best estimate of the true number of classes for a CNVR. The best-performing cohort was the cohort in which the clusters were most clearly defined and hence in which the highest number of polymorphic CNVRs could be called. In this way, where the number of classes initially clustered in the first run of the algorithm was different across cohorts, we re-clustered CNVRs, fixing the number of classes to be that obtained in the first run in the best-performing cohort. CNVRs were determined to be high quality if they were called in the best-performing cohort or had class frequencies compatible with the best-performing cohort (Additional file 1).

For stage 2, lung function measurements (FEV_1_, FVC and FEV_1_/FVC) were available for 3 adult cohorts (BHS, B58C and SHIP) and 1 child cohort (ALSPAC). We tested for association of FEV_1_, FVC and FEV_1_/FVC with CNVR copy number within each cohort and results were then meta-analysed across up to 4 cohorts as the primary analysis and then across up to 3 cohorts of adults to look for age specific effects. Only CNVRs called with the same number of classes and consistent class frequencies across cohorts in stage 1 (Additional file 1) were included in meta-analyses. Uncertainty in copy number assignment due to weakly defined clustering was taken into account in association testing by using copy number dose (Additional file 1) [20]. Association testing for cohorts with unrelated individuals was performed using a linear model of phenotype with copy number dose. Lung function phenotypes were adjusted for age, age^2^, sex, height and height^2^ and first 4 ancestry principal components. As there were related individuals in BHS we used a generalised-estimating equation (GEE) to account for correlation within families [21]. A detailed description of the cohorts is given in the Supplementary Material. A flow diagram of the whole pipeline from all initially available samples and CNVRs through to final meta-analysis results is shown in Additional file 1: Figure S1.

For CNVRs which were well-tagged by SNPs (r^2^ > 0.7), we sought replication of suggestive signals of association (*P* < 0.001) for lung function in a large GWAS. Linkage disequilibrium (r^2^) between each CNVR and SNPs within 1 Mb of the CNVR start and end positions was measured using samples in the BHS cohort that had both copy number genotypes and imputed SNP genotypes (1000 Genomes Project Phase 1 imputation reference panel). For replication we used results from the SpiroMeta and CHARGE consortia meta-analysis of 48,201 individuals across 23 studies [3, 4], the UK BiLEVE lung function GWAS [6] (*n* = 48,943, Additional file 1) and the subset of samples from the 152,729 UK Biobank samples genotyped at the time of writing that were not in UK BiLEVE and with lung function measurements passing ERS/ATS criteria (*n* = 49,727, Additional file 1). In the replication studies lung function traits were adjusted for age, age^2^, sex, height, pack years in smokers where available, and ancestry principal components where available. Residuals were inverse rank inverse-normal transformed after adjustment apart from FVC in SpiroMeta and CHARGE, which was untransformed [4], hence for FEV_1_ and FEV_1_/FVC we used inverse-variance weighted meta-analysis across the replication cohorts and for FVC we used Z-score meta-analysis. All replication resources were comprised of European ancestry individuals. For replication, we used a Bonferroni corrected 5 % threshold for the number of CNVRs with a tag SNP available. Replication was not available for CNVRs not well-tagged by SNPs (r^2^ > 0.7).

We tested for enrichment of gene ontology (GO) terms using DAVID [http://david.abcc.ncifcrf.gov/] within genes spanned by CNVRs showing association with *P* < 0.01 for our lung function traits in the meta-analysis of all cohorts and separately within the meta-analysis of adult and child cohorts. We used a Bonferroni correction for all GO terms showing nominal significance of *P* < 0.05. We used GTEx[22] to perform a look up of eQTL signals of SNPs tagging CNVRs using an empirical 5 % threshold determined by permutation by GTEx.

## Results

For the first stage of the analysis, aimed at developing a pipeline for copy number calling across different genotyping platforms, intensity data was available for 24,259 samples from 6 cohorts. After filtering, 19,308 samples were taken forward for CNVR clustering and copy number calling. The total number of CNVRs called and the proportions of deletions, amplifications and multiallelic CNVRs called in each cohort varied across genotyping platforms (Table 1) and hence we only meta-analysed CNVRs that were called consistently across platforms. As CNVCALL was able to resolve the largest proportion of the 2788 CNVRs as polymorphic in the BHS cohort data (1962 CNVRs, 70.4 %) and the clustering gave the best separation of classes in the BHS data (Additional file 1), the number of classes called in the BHS cohort was used as our best estimate of the true number of classes and as the standard for calling CNV genotypes with a consistent number of classes across all cohorts. The numbers of CNVRs initially clustered in the 6 cohorts are shown in Table 1 and the overlap of clustered CNVRs across cohorts is shown in Additional file 1: Figure S2. Differences in the numbers of CNVRs successfully clustered in each cohort could be due to differences in data quality, differences in probe content on different genotyping platforms or the available intensity measures (LRR vs X + Y). 1103 CNVRs could be clustered with number of classes and class frequencies compatible with BHS (χ^2^ test described in Additional file 1) in at least 1 other cohort. This filtered set of CNVRs was enriched for deletions in all cohorts compared to the initial set (Table 1).

**Table 1:** Platform probes, samples and CNVRs clustered

Cohort	BHS	YFS	B58C	SHIP	Raine	ALSPAC
Platform	Illumina 660	Illumina 670	Illumina 1.2 M	Affymetrix 6	Illumina 660	Illumina 550
Autosomal SNP probes	573462	580030	1115905	909508	578525	580694
CNV probes^a^	62092	63617	75114	945805	62138	0
Samples used in CNVR clustering	3496	2682	2920	4072	1685	9382
No. CNVRs clustered within cohort	1962	1933	1540	721	1929	491
Percentage of deletion/amplification/multiallelic	43.9/34.0/22.1	44.0/33.9/22.1	46.1/34.0/19.9	38.6/40.7/20.7	44.0/34.0/22.0	43.6/40.5/16.0
No. CNVRS consistently clustered with BHS	1962	393	855	224	838	11
Percentage of deletion/amplification/multiallelic	43.9/34.0/22.1	52.7/24.7/22.6	51.3/27.8/20.8	57.6/23.2/19.2	52.5/28.3/19.2	72.7/27.3/0

^a^A CNV probe is a monomorphic probe targeted in regions of known copy number variation

For the second stage of the analysis, lung function data was available for 19,870 samples from 4 cohorts. After filtering, 12,403 samples were taken forward for association testing (Table 2). None of our CNVR associations reached a 5 % Bonferonni threshold for 1962 independent tests (*P* < 2.5 × 10^-5^); the full set of association results are provided in Additional file 2. We sought replication of suggestive associations (*P* < 0.001) which included 12 CNVR-trait combinations (11 distinct CNVRs) in the adult cohorts analysis (Table 3; Quantile-Quantile and Manhattan plots are in Additional file 1: Figure S4 and Figure S5).

**Table 2:** Summary of samples with lung function phenotypes

Cohort	BHS	B58C	SHIP	ALSPAC
Samples passing QC (M/F)	3084 (1381/1703)	2492 (1293/1199)	1765 (863/902)	5062 (2547/2515)
Age yrs mean (range)	50.0 (16.5–97.3)	45.1 (44.5–46.0)	52.3 (25.0–85.0)	8.64 (7.42–10.33)
Height m mean (range)	1.69 (1.39–1.97)	1.70 (1.22–2.02)	1.70 (1.42–1.97)	1.33 (1.13–1.59)
FEV_1_ L mean (range)	3.09 (0.56–6.90)	3.30 (0.65–5.73)	3.28 (0.88–6.32)	1.7 (0.68–2.79)
FVC L mean (range)	3.96 (0.96–8.63)	4.22 (1.1–7.71)	3.86 (1.22–7.24)	1.93 (0.77–3.13)
FEV_1_/FVC mean (range)	0.78 (0.28–1.00)	0.79 (0.12–1.00)	0.85 (0.49–1.00)	0.88 (0.50–1.00)

**Table 3:** CNV association with lung function with *P* < 0.001 for meta-analysis of 3 adult cohorts with follow up of tag SNPs in replication cohorts

	CNVR	CNV results					Replication^c^										
		Meta analysis of BHS, B58C & SHIP					Best tag SNP		SpiroMeta & CHARGE		UK BiLEVE		UK Biobank		Meta-analysis^d^		
		N	Beta L (SE)	Effect^b^	P	Genes	SNP	r^2^	Beta (SE)	P	Beta (SE)	P	Beta (SE)	P	Beta (SE)	Effect^e^	P
FEV_1_	CNVR1073.1 (2q32.1) 640 bp deletion (2 class)	5560	-0.0467 (0.0135)	--.	5.55×10^-4^	Upstream *DNAJC10*	rs2696127	0.766	-0.0069 (0.008)	0.38	-0.0073 (0.007)		-0.0061 (0.007)	0.37	0.0068 (0.004)	----	0.11
	CNVR217.1^a^ (1p31.1) 45.7kbp multiallelic (3 class)	7258	0.0268 (0.0078)	+++	5.55×10^-4^	Upstream *NEGR1*	rs2568958	1	0.0084 (0.007)	0.24	0.0040 (0.007)	0.56	0.0072 (0.007)	0.27	-0.0065 (0.004)	++++	0.10
	CNVR4222.1^a^ (9p21.2) 4.01kbp deletion (2 class)	7284	-0.0868 (0.0263)	---	9.78×10^-4^	Intronic *LINGO2*	rs10968307	0.980	0.0067 (0.031)	0.83	-0.0129 (0.026)	0.61	0.0084 (0.024)	0.73	0.0003 (0.015)	- + -+	0.99
FVC	CNVR4742.1 (10q21.1) 1.35kbp deletion (3 class)	5528	0.0599 (0.0174)	++.	5.65×10^-4^	intergenic	rs1903969	0.995	0.0020 (0.005)	0.72	0.0082 (0.010)	0.40	0.0048 (0.009)	0.61		++++	0.32
	CNVR4222.1^a^ (9p21.2) 4.01kbp deletion (2 class)	7284	-0.1126 (0.0329)	---	6.30×10^-4^	Intronic *LINGO2*	rs10968307	0.980	0.0342 (0.106)	0.75	-0.0150 (0.026)	0.56	-0.0126 (0.024)	0.60		- + --	0.65
	CNVR6854.1^a^ (16q24.2) 3.25kbp deletion (3 class)	7281	0.0389 (0.0116)	+++	7.72×10^-4^	Downstream *BANP*	rs7501378	0.940	0.0111 (0.005)	0.017	0.0232 (0.008)	0.0037	-0.0021 (0.008)	0.79		+++-	0.0038
	CNVR7142.1^a^ (17q22) 2.62kbp deletion (3 class)	5519	-0.0454 (0.0136)	--.	8.20×10^-4^	Downstream *SCPEP1*	rs880266	0.766	-0.0021 (0.006)	0.73	-0.0048 (0.008)	0.53	0.0108 (0.007)	0.14		---+	0.77
FEV_1_ / FVC	CNVR94.3^a^ (1p36.13) 14.5kbp deletion (3 class)	4800	0.0061 (0.0016)	+.-	1.96×10^-4^	Upstream *CROCC*	rs696095	0.263									
	CNVR94.5 (1p36.13) 51.3kbp deletion (4 class)	3005	0.0045 (0.0012)	+..	2.08×10^-4^	Exonic *CROCC*	rs696095	0.169									
	CNVR94.4 (1p36.13) 106kbp multiallelic (4 class)	2951	0.0046 (0.0013)	+..	2.69×10^-4^	Exonic *CROCC*	rs696095	0.169									
	CNVR3585.1 (7q34) 560 bp multiallelic (4 class)	3082	-0.0052 (0.0014)	-..	2.89×10^-4^	Intronic *MGAM*	rs62477625	0.644									
	CNVR7927.1^a^ (20q13.33) 880 bp gain (3 class)	5548	0.0056 (0.0016)	++.	5.98×10^-4^	upstream *HAR1A*; downstream *HAR1B*; downstream *LOC63930*	rs4809276	0.818	-0.0037 (0.010)	0.71	0.0083 (0.008)	0.30	0.0056 (0.008)	0.47	-0.0044 (0.005)	+-++	0.37

*FEV*_*1*_ Forced Expiratory Volume in 1 second, *FVC* Forced Vital Capacity (FVC). ^a^CNVR class frequencies consistent with independent cohort (Additional file 1: Table S1). ^b^Effect direction: BHS/B58C/SHIP (a dot signifies no result). r^2^ correlation coefficient between CNVR copy number and SNP genotype in BHS. Replication was performed where there was a tag SNP with r^2^ > 0.7. ^c^Lung function traits are rank inverse-normalised apart from FVC in SpiroMeta & CHARGE which is untransformed mL. ^d^Inverse-variance weighted meta-analysis for FEV_1_ and FEV_1_/FVC; Z-score meta-analysis for FVC. ^e^Effect direction: CNVR/SpiroMeta & CHARGE/UK BiLEVE/UK Biobank. Gene annotations provided by ANNOVAR [30]

Replication was sought for these 12 CNVR associations by looking at association of SNPs tagging the CNVR in independent cohorts (Table 3). Out of 12 CNVR-trait combinations showing suggestive evidence of association with lung function, (*P* < 0.001), 7 (including CNVR4222.1 which was associated with both FEV_1_ and FVC; Table 3) were well tagged by a 1000G SNP (r^2^ > 0.7). A SNP tagging CNVR6854.1 (rs7501378, r^2^ = 0.94) showed replication of association with FVC in UK BiLEVE and in the meta-analysis of the 3 replication cohorts (Table 3) using a Bonferroni-corrected 5 % threshold for 8 tests (*P* < 0.006). The direction of effect of this CNVR was consistent across the 3 discovery CNVR cohorts and was in the same direction in SpiroMeta, CHARGE and UK BiLEVE for the SNP allele positively correlated with increased copy number, but in the opposite direction in the non-significant UK Biobank association (Table 3). CNVR6854.1 is located at 16q24.2 and is 2.0 kb downstream of *BANP* (BTG3 Associated Nuclear Protein, also known as Scaffold/Matrix-Associated Region-1-Binding Protein [*SMAR1*]). The tag SNP rs7501378 is 420 bp further downstream of *BANP* than CNVR6854.1 (Additional file 1: Figure S6). There was no evidence for this SNP as an eQTL in blood [23, 24] or lung [24].

The 3 strongest signals of association with lung function in the discovery meta-analysis (FEV_1_/FVC, *P* = 1.96-2.69 × 10^-4^, CNVR94.3, CNVR94.4 and CNVR94.5) were for 3 overlapping and correlated CNVRs within or near to *CROCC*, a gene that encodes rootletin, a component of cilia (Additional file 1: Figure S7). CNV94.3 was measured as a 3 class CNV in a previous study [17] and showed consistent class frequencies with those observed in BHS and B58C (Additional file 1: Table S1). However, no tagging SNP was available.

Across the 45 genes implicated by CNVRs showing nominal evidence of association (*P* < 0.01) with lung function in this study, no enrichment of gene ontology terms was seen (*P* < 0.05).

## Discussion

We developed a quality-control pipeline for genome-wide association analysis and meta-analysis of CNV genotypes and generated robust copy number calls across cohorts for 1962 CNVs genome-wide across 6 cohorts comprising 5 different genotyping platforms. Of these, 1103 CNVs could be clustered consistently in at least 2 cohorts, 777 in 3 cohorts, 371 in 4 cohorts and 70 in 5 cohorts. No CNVRs could be clustered across all 6 cohorts (Additional file 1: Figure S2). We tested for association of each CNVR with lung function in a subset of 4 cohorts and then undertook meta-analyses for adults and children combined and adults only. A signal of association with FVC for a CNVR downsream of *BANP* on chromosome 16 was replicated using a tag SNP in 2 large independent studies. The class frequencies of this CNVR observed in our study were consistent with those previously measured [17] providing reassurance that this CNVR has been measured accurately. *BANP* is expressed in a range of organ tissues including respiratory epithelial cells [http://www.proteinatlas.org/] and has been shown to have a role in regulation of alternative splicing via a histone deactylase 6 (HDAC6)-mediated deactylation pathway [25, 26] (other HDACs, including HDAC6, have previously been implicated in chronic obstructive pulmonary disease (COPD) and lung function [27, 28]).

An additional five CNVRs which showed suggestive evidence of association with lung function in our study were complex (i.e. did not have 3 copy number classes) and hence not well tagged by bi-allellic 1000G SNPs. Amongst these were 3 correlated CNVRs which had the strongest evidence of association for FEV_1_/FVC (*P* = 2.0-2.7 × 10^-4^) and which involved the *CROCC* gene in a region that has previously been shown to be associated with lung function [3]. These 3 CNVRs are not tagged (r^2^ = 0.005) by the previously reported SNP showing strong evidence of association with FEV_1_/FVC (rs2284746, *P* = 7.5x10^-16^), which is within 25 kb [3]. Collectively, these 3 CNVRs would explain an additional 0.46 % of the variance in FEV_1_/FVC in addition to the 3.2 % of variance in FEV_1_/FVC already accounted for by genome-wide significantly associated SNPs [3]. It has been shown in *Crocc* knockout mice that loss of rootletin (the protein encoded by *Crocc*) prevents the formation of the ciliary rootlet in airway epithelial cells leading to a reduction in motile cilium function with pathological changes consistent with insufficient mucociliary clearance [29]. *CROCC* is in a region which is annotated as containing several overlapping CNVRs [17] (Additional file 1: Figure S7); however some nested CNVRs could actually be the result of incomplete measurement of the larger CNVR within which they are found. The preliminary CNV associations in *CROCC* warrant further verification. Determining the precise genomic architecture of the region is needed to understand the nature of the associations observed in this study.

Meta-analysis of association test statistics from multiple cohorts is a routine approach to increase sample sizes in GWAS based on SNP data when individual-level data cannot be analysed all together. SNPs that are assayed on commercial platforms are biallelic and well characterised in terms of expected allele frequencies and the assays themselves are designed to accurately measure SNP genotypes. In contrast, CNVRs are less well-characterised and although the use of SNP genotyping platform intensity presents a useful and economical approach to measure copy number variation, this also presents challenges. In this study, we presented a pipeline for ensuring compatibility of CNV genotypes before meta-analysis when these have been derived from different data sources (both in terms of platform and probe set). We were able to generate compatible copy number calls across at least 2 cohorts for over one thousand CNVRs genome-wide. Differences in concordance of CNV class frequencies between pairs of cohorts with SNP genotyping data from the same or similar versions of Illumina platforms could be due to differences in intensity data quality. It was evident that older versions of the platforms had poorer probe coverage of the CNVRs than the more recent platforms, some of which had content designed specifically for the larger set of CNVRs from which the subset analysed here were selected. We chose to use the number of copy number classes and class frequencies from one cohort as the best estimate of the true number of classes to which other cohorts were compared with validation of the genotypes measured in this reference cohort against CNV genotypes in an independent cohort. As new cohorts emerge and existing cohorts update their genotype data using newer arrays, compatibility across cohorts for increasing numbers of CNVRs is likely to increase.

## Conclusions

We demonstrate how common CNV regions can be reliably and consistently called across cohorts, using an existing calling algorithm and rigorous quality control steps, using SNP genotyping array intensity data. Using this approach, we describe a novel signal of association with FVC for a copy number variable locus downstream of *BANP* on chromosome 16 and present evidence that copy number variation may play a role at a locus previously reported as being associated with lung function (*CROCC*).

## Abbreviations

1000G, 1000 genomes project; ALSPAC, avon longitudinal study of parents and children; B58C, British 1958 birth cohort; BHS, busselton health study; CHARGE, cohorts for heart and aging research in genomic epidemiology; CNV, copy number variant; CNVR, copy number variable region; ERS/ATS, european respiratory society / american thoracic society; FEV_1_, forced expiratory volume in 1 second; FVC, forced vital capacity; GWAS, genome-wide association study; LRR, Log R ratio; SHIP, study of health in pomerania; SNP, single nucleotide polymorphism; UK BiLEVE, UK biobank lung exome Variant Evaluation; YFS, young finns study

## Additional files

Additional file 1: Supplementary methods, description of studies, supplementary tables and figures. (PDF 913 kb) Additional file 2: Copy number class frequencies and lung function association results (FEV_1_, FVC and FEV_1_/FVC) for all 1962 CNVRs that were tested. (XLSX 1019 kb)
