# Supplementary material for: Genome-wide association study of copy number variation with lung function identifies a novel signal of association near BANP for forced vital capacity
Source: BMC Genet. 2016 Aug 11;17:116. doi: 10.1186/s12863-016-0423-0 (PMC4981989; doi:10.1186/s12863-016-0423-0)
Supplement: Additional file 1: — Supplementary methods, description of studies, supplementary tables and figures. (PDF 913 kb) [file 12863_2016_423_MOESM1_ESM.pdf]

Supplementary Material

Supplementary Methods

Selection of CNVRs

Of the 3276 autosomal CNVs from the Genome Structural Variation Consortium [1], 45 were deemed to be likely artefacts that had arisen due to cross-hybridization of probes between an autosome and a sex-chromosome and 71 were deemed to be artefacts of cell line somatic rearrangements at the immunoglobulin locus. Of the remaining autosomal 3160, 372 were found to be redundant (i.e. identified as multiple overlapping CNVs in the discovery experiment but subsequently found to represent one CNV) and were excluded (for a further 1289 CNVs, redundancy could not be determined; these were retained for this analysis).

Pre-clustering sample quality control for intensity signal noise

Variation in the level of noise from the intensity signal Log R Ratio (LRR) has a more profound effect on the accuracy of copy number genotyping than on the accuracy of SNP genotyping. The B allele frequency (BAF) is the allelic intensity ratio of the centres of the 3 genotype clusters [2]. Figure S10 shows the BAF standard deviation per sample plotted against the LRR standard deviation for each sample. It has been found that high BAF standard deviation is indicative of increased sample noise which leads to an significant increase in spurious copy number calls when using CNV discovery algorithms that call CNVs on a per-sample basis [3]. Therefore, for CNV discovery, samples with BAF sd > 0.1 were excluded (Figure S10); no LRR threshold was applied. In contrast to the other cohorts for which Illumina genotype platforms were used, the SHIP cohort data was derived from the Affymetrix Genome-Wide Human SNP Array 6.0. A higher BAF sd exclusion threshold of 0.12 was chosen for SHIP as a threshold of 0.1 was found to be over conservative. For ALSPAC, only normalised X and Y intensity data were available for this study. No ALSPAC samples were excluded based on a plot of X intensity standard deviation against Y intensity standard deviation that showed no obvious outlying samples (Figure S11).

For each CNVR, samples which had missing data for any of the probes within that CNVR were excluded. CNVRs were excluded from analysis for individual cohorts if more than 50% of samples in the cohort were excluded after applying the sample exclusion above. Clustering was not possible for some CNVRs

in some cohorts due to there being no probes present on the platform within the CNVR.

Clustering procedure

For each CNVR, four alternative 1-dimensional summaries of the LRRs (or X+Y for ALSPAC) across the probes within the CNVR boundary were calculated for each sample [4]. These were: (i) the mean; (ii) mean with linear discriminant function (LDF) that maximises the correlation between summary value and posterior genotype probability (iii); 1st principal component (PCA); (iv) PCA with LDF. The samples were then clustered using each summary value at each CNVR by Bayesian hierarchical mixture modelling implemented in the R package CNVCALL [3, 4]. The average posterior probability (APP) across samples of their most likely genotypes was used as a measure of how well the intensity data had been clustered and accordingly indicated the reliability of genotype calls for the CNVR. Supplementary Figure S3 gives examples of strong and weak clustering. APP was not used to exclude CNVRs from further analysis – uncertainty in copy number assignment due to weakly defined clustering was taken into account in association testing by using copy number dose.

The 1-dimensional summary method (mean, mean with LDF, PCA or PCA with LDF) that gave the highest number of copy number classes was chosen unless more than one method produced the maximum number of classes, in which case the method giving the highest average posterior probability (APP) was chosen [4]. CNVRs were called as missing for any cohort where there were no probes within the CNVR on the genotyping platform or if CNVCALL was unable to resolve the intensity data into more than 1 copy number class (indicating that either the method is insensitive to copy number variation at that particular CNVR or that there is no copy number variation in the cohort at that CNVR).

For 50 CNVRs, chosen randomly from those for which different numbers of classes were called in different cohorts, we visually inspected the clustering histograms and also scatter plots of intensity values against phenotypes in order to compare the distinction of copy number classes in each cohort.

Consistency of copy number class frequencies across cohorts

We checked for consistency of clustering across cohorts by comparing the class frequencies between cohorts at each CNVR. We calculated the statistic for a χ^2 test of frequencies between those observed in BHS and each of the other cohorts (pairwise, each separately with BHS). We defined class frequencies as

compatible if the χ^2 test statistic < 20 (P ~ 7.7×10^{-6}). This corresponds to a Bonferroni corrected 5% level for approximately 6500 independent tests. Using this criterion, any cohort with a significant difference in class frequencies was excluded for that CNVR. At each CNVR showing evidence of association, any cohorts with low clustering quality (APP < 0.95; threshold based on visual inspection of cluster histograms) were flagged and the clustering histograms were re-checked by eye.

Dose calculation

Copy number dose is on a continuous scale reflecting the uncertainty in copy number assignment e.g. for a 3 class CNVR the dose is continuous from 0 to 2. CNVCALL determines the number of copy number classes k corresponding to the number of intensity signal clusters and for each sample returns k posterior probabilities of the sample belonging to each copy number $p_1, p_2 \ldots p_k$. CNVCALL assigns the remaining probability to a “null” class corresponding to the sample not belonging to any class. The probability of null class is $p_{null} = 1 - \sum_{i=1}^{k} p_i$. The steps for calculating the dose are:

1. If $\sum_{i=1}^{k} p_i < 0.5$ discard the CNVR (i.e. if the null class is more probable than the others)

2. Rescale the non-null probabilities so that they add to 1:

   $p_i^* = \frac{p_i}{\sum_{i=1}^{k} p_i}$ e.g. A 2 class CNVR with probabilities 0.2, 0.6 (0.2 null class) becomes:

   0.2/0.8, 0.6/0.8 = 0.25, 0.75

3. Calculate dosage from rescaled probabilities:

   $Dose = \sum_{i=1}^{k} p_i^* (i-1)$ e.g. for a 3 class CNVR with rescaled non-null probabilities

   (0.1, 0.7, 0.2) the dose is $(0.1\times0) + (0.7\times1) + (0.2\times2) = 1.1$

Proportion of variance explained

The proportion of variance explained by a CNVR is given by the coefficient of determination (R^2) which is approximated by:

$$R^2 = \frac{\left(\frac{beta}{se}\right)^2}{n-1}$$

Replication in UK BiLEVE

The UK Biobank Lung Exome Variant Evaluation study (UK BiLEVE) was the first genetic study in UK Biobank [5]. UK Biobank (http://www.ukbiobank.ac.uk/) contains data from 502,682 individuals (94% of self-reported European ancestry) with extensive health and life-style questionnaire data, physical measures (including spirometry) and DNA. Spirometry was undertaken using a Vitalograph Pneumotrac 6800. The participant was asked to record two to three blows (lasting for at least 6 seconds) within a period of about 6 minutes. The computer compared the reproducibility of the first two blows and, if acceptable (defined as a <5% difference in forced volume vital capacity (FVC) and Forced Expiratory Volume in 1 second (FEV_1), a third blow was not required. For the UK BiLEVE project, a sampling frame of 275,939 individuals was defined as those who were of European ancestry and had spirometry measures which met ERS/ATS guidelines[6]. A total of 50,008 samples were selected from the extremes and middle of the distributions of percent predicted FEV_1, separately in never smokers and heavy smokers (20,005 individuals with low FEV_1, 19,997 with average FEV_1 and 10,006 with high FEV_1). DNA was extracted and genotyped with the custom-designed Affymetrix Axiom UK BiLEVE array. Following thorough variant and sample QC, 48,943 unrelated individuals remained for further analysis.

We performed a look up for replication in the results of the meta-analysis of lung function traits (FEV_1, FVC and FEV_1/FVC) across smoking strata.

Replication in UK Biobank

There were 51,117 samples available in UK Biobank with spirometry measures (described above) which met ERS/ATS guidelines but were not selected for the UK BiLEVE study. After excluding 41 samples with recorded sex inconsistent with genotype data, 124 samples of non-white ancestry and 1225 samples related either to other UK BiLEVE or UK Biobank samples, there remained 49,727 samples available for replication, 31,952 smokers and 17,775 non-smokers. Association results of lung function traits (FEV_1, FVC and FEV_1/FVC) were meta-analysed across smoking strata.

Description of Studies

Busselton Health Study (BHS) is a series of cross sectional population health studies in Busselton in South Western Australia undertaken between 1966 and 1990. Blood for DNA extraction was taken in 1994-95. Spirometric measures of FEV_1 and FVC have been described previously [7].

The Busselton Health Study genetics study is approved by the University of Western Australia Human Ethics Committee under project numbers RA/4/1/1516 and RA/4/1/2077. Access to Busselton data was approved by the Research Committee for The Busselton Population Medical Research Foundation (approval number SN10/06).

The British 1958 Birth Cohort (B58C) is a national population-based cohort followed up from birth to age 44-45. Spirometry was also performed at age 44-45 in a standing position. Phenotypes are detailed online (http://www.b58cgene.sgul.ac.uk/phenomenu.php).

The British 1958 birth cohort biomedical follow-up (2002-2004), which included the DNA collection on which all subsequent genetic association studies have been based, was approved by the South East England Multi-Centre Research Ethics Committee (reference: MREC/01/1/44). Access to British 1958 Birth Cohort data was approved by the British 1958 Birth Cohort Access Committee/Access Committee for CLS Cohorts (approval: 2009_DATA_05_Wain).

The Study of Health in Pomerania (SHIP) is a survey of the adult population from West Pomerania in Germany (28). SHIP-0, the baseline study, comprising women and men aged 20 to 79 years was drawn between October 1997 and May 2001; whereas SHIP-1, the first 5-year-follow-up was conducted between October 2002 and September 2006. Spirometry measures (SHIP-1) were taken seated 3 times in order to obtain at least 2 reproducible values.

The Study of Health in Pomerania was approved by the local ethics committee of the University of Greifswald. Access to Study of Health in Pomerania data were via the Community Medicine Research Network, Department for Management and Transfer of Data and Biomaterials of the University of Greifswald (http://www.medizin.uni-greifswald.de/icm/transferstelle/).

The Avon Longitudinal Study of Parents and Children (ALSPAC) recruited 14,541 pregnant women

resident in Avon, UK with expected dates of delivery 1st April 1991 to 31st December 1992. 14,541 is the initial number of pregnancies for which the mother enrolled in the ALSPAC study and had either returned at least one questionnaire or attended a “Children in Focus” clinic by 19/07/99. Of these initial pregnancies, there was a total of 14,676 foetuses, resulting in 14,062 live births and 13,988 children who were alive at 1 year of age.

When the oldest children were approximately 7 years of age, an attempt was made to bolster the initial sample with eligible cases who had failed to join the study originally. As a result, when considering variables collected from the age of seven onwards (and potentially abstracted from obstetric notes) there are data available for more than the 14,541 pregnancies mentioned above.

The number of new pregnancies not in the initial sample (known as Phase I enrolment) that are currently represented on the built files and reflecting enrolment status at the age of 18 is 706 (452 and 254 recruited during Phases II and III respectively), resulting in an additional 713 children being enrolled. The phases of enrolment are described in more detail in the cohort profile paper [8].

The total sample size for analyses using any data collected after the age of seven is therefore 15,247 pregnancies, resulting in 15,458 foetuses. Of this total sample of 15,458 fetuses, 14,775 were live births and 14,701 were alive at 1 year of age.

Spirometry was performed using the Vitalograph Spirotrac IV system (Vitalograph, Maids Moreton UK) and the hand-held Medikro Spirostar USB spirometer (Medikro, Kuopio, Finland) using methods described previously [9, 10]. The machines were calibrated every day the medical examination took place. FVC and FEV1 were measured in sitting position, while wearing a nose clip, by trained personnel, according to the ATS/ERS guidelines. For each child, at least three acceptable manoeuvres had to be obtained. The best results of three acceptable & repeatable (FVC +/- 150mL) flow-volume curves were accepted after post hoc quality control by a respiratory physician.

Please note that the ALSPAC study website contains details of all the data that is available through a fully searchable data dictionary (http://www.bris.ac.uk/alspac/researchers/data-access/data-dictionary/).

Ethical approval for the Avon Longitudinal Study of Parents and Children (ALSPAC) was obtained from

the ALSPAC Ethics and Law Committee and the Local Research Ethics Committees. All data provided for the analyses was anonymised and no patient identifying information was held by the central analysis group. Access to the Avon Longitudinal Study of Parents and Children was approved by the ASLPAC Executive Committee (approved project B1186, http://www.bristol.ac.uk/alspac/researchers/data-access/).

Cardiovascular risk in Young Finns Study (YFS) was a study comprising 3500 children and adolescents recruited in 1980. Lung function was not measured.

The Young Fins Study has been approved by the Ethics Committee of the South-western Finland Hospital District. Access to Young Finns data was approved by the Young Finns Study group.

The Raine Study is a cohort of children formed in 1989-91 when 2900 pregnant women volunteered to be part of the study at King Edward Memorial Hospital in Perth, Australia. Lung function was not available.

Recruitment to the Raine Study and all follow-ups were approved by the Human Ethics Committee at King Edward Memorial Hospital and/or Princess Margaret Hospital for Children (PMH). Access to the Raine data was approved by the Raine Study Executive Committee.

References

1. Conrad DF, Pinto D, Redon R, Feuk L, Gokcumen O, Zhang Y, Aerts J, Andrews TD, Barnes C, Campbell P, Fitzgerald T, Hu M, Ihm CH, Kristiansson K, MacArthur DG, MacDonald JR, Onyiah I, Pang AWC, Robson S, Stirrups K, Valsesia A, Walter K, Wei J, Tyler-Smith C, Carter NP, Lee C, Scherer SW, Hurles ME: **Origins and functional impact of copy number variation in the human genome**. *Nature* 2010, **464**:704–712.

2. Peiffer DA, Le JM, Steemers FJ, Chang W, Jenniges T, Garcia F, Haden K, Li J, Shaw CA, Belmont J, Cheung SW, Shen RM, Barker DL, Gunderson KL: **High-resolution genomic profiling of chromosomal aberrations using Infinium whole-genome genotyping**. *Genome Research* 2006, **16**:1136–1148.

3. Cardin N, Holmes C, The Wellcome Trust Case Control Consortium, Donnelly P, Marchini J: **Bayesian hierarchical mixture modeling to assign copy number from a targeted CNV array**. *Genet Epidemiol* 2011, **35**:536–548.

4. Craddock N, Hurles ME, Cardin N, Pearson RD, Plagnol V, Robson S, Vukcevic D, Barnes C, Conrad DF, Giannoulatou E: **Genome-wide association study of CNVs in 16,000 cases of eight common diseases and 3,000 shared controls**. *Nature* 2010, **464**:713–720.

5. Wain LV, Shrine N, Miller S, Jackson VE, Ntalla I, Soler Artigas M, Billington CK, Kheirallah AK, Allen R, Cook JP, Probert K, Obeidat M 'en, Bossé Y, Hao K, Postma DS, Paré PD, Ramasamy A, Mägi R, Mihailov E, Reinmaa E, Melén E, O'Connell J, Frangou E, Delaneau O, Freeman C, Petkova D, McCarthy M, Sayers I, Deloukas P, Hubbard R, et al.: **Novel insights into the genetics of smoking behaviour, lung function, and chronic obstructive pulmonary disease (UK BiLEVE): a genetic association study in UK Biobank**. *The Lancet Respiratory Medicine* , **3**:769–781.

6. Miller MR, Hankinson J, Brusasco V, Burgos F, Casaburi R, Coates A, Crapo R, Enright P, van der Grinten CPM, Gustafsson P, Jensen R, Johnson DC, MacIntyre N, McKay R, Navajas D, Pedersen OF, Pellegrino R, Viegi G, Wanger J: **Standardisation of spirometry**. *European Respiratory Journal* 2005, **26**:319–338.

7. James AL, Palmer LJ, Kicic E, Maxwell PS, Lagan SE, Ryan GF, Musk AW: **Decline in Lung Function in the Busselton Health Study**. *Am J Respir Crit Care Med* 2005, **171**:109–114.

8. Boyd A, Golding J, Macleod J, Lawlor DA, Fraser A, Henderson J, Molloy L, Ness A, Ring S, Davey Smith G: **Cohort Profile: The "Children of the 90s"—the index offspring of the Avon Longitudinal Study of Parents and Children**. *International Journal of Epidemiology* 2012.

9. Kotecha SJ, Watkins WJ, Heron J, Henderson J, Dunstan FD, Kotecha S: **Spirometric lung function in school-age children: effect of intrauterine growth retardation and catch-up growth.** *Am J Respir Crit Care Med* 2010, **181**:969–974.

10. Cremers E, Thijs C, Penders J, Jansen E, Mommers M: **Maternal and child's vitamin D supplement use and vitamin D level in relation to childhood lung function: the KOALA Birth Cohort Study**. *Thorax* 2011.

Supplementary Tables

Table S1: Comparison of CNV copy number class frequencies observed in BHS in this study compared to frequencies observed in HapMap samples by Conrad *et al.* **[1]** for 6 of our 11 reported CNVRs where HapMap frequencies were available.

CNVR	BHS observed genotypes in this study (3496 samples)	HapMap observed genotypes (180 samples)	Fisher test P-value
CNVR217.1	1245/1632/581	76/77/25	0.19
CNVR4222.1	132/3323	7/170	0.84
CNVR6854.1	150/1192/2114	7/59/110	0.97
CNVR7142.1	213/1273/1961	6/70/102	0.29
CNVR94.3	1064/1673/719	57/82/26	0.25
CNVR7927.1	2318/1027/122	114/47/4	0.78

Supplementary Figures

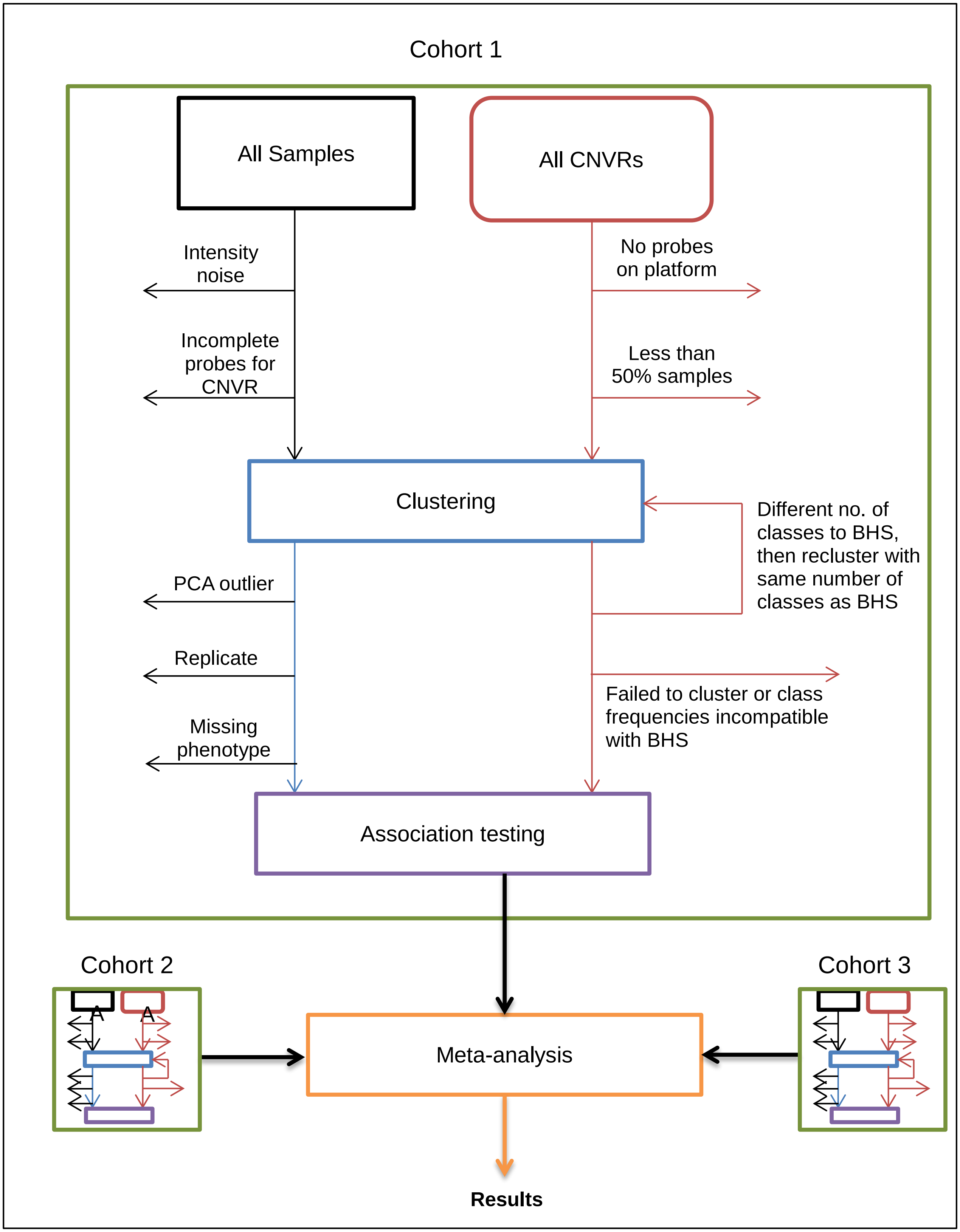

Figure S1: Flow diagram of analysis pipeline

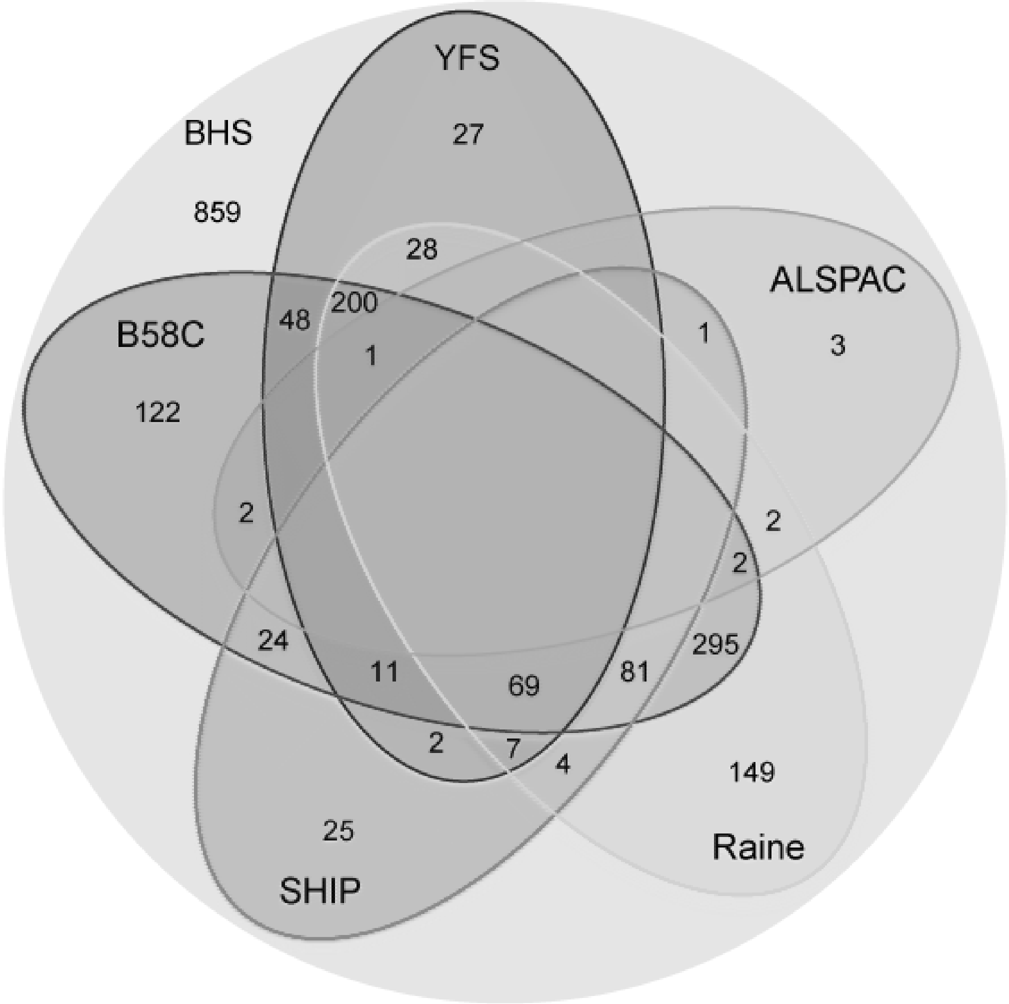

Figure S2: Venn diagram showing numbers of CNVRs that had compatible class frequencies with BHS in each cohort. 1962 CNVRs in total successfully clustered in BHS, 855 in B58C, 224 in SHIP, 11 in ALSPAC, 393 in YFS and 838 in Raine. 1103 CNVRs had class frequencies compatible with BHS and at least 1 other cohort, 859 did not have class frequencies compatible with BHS in any other cohort.

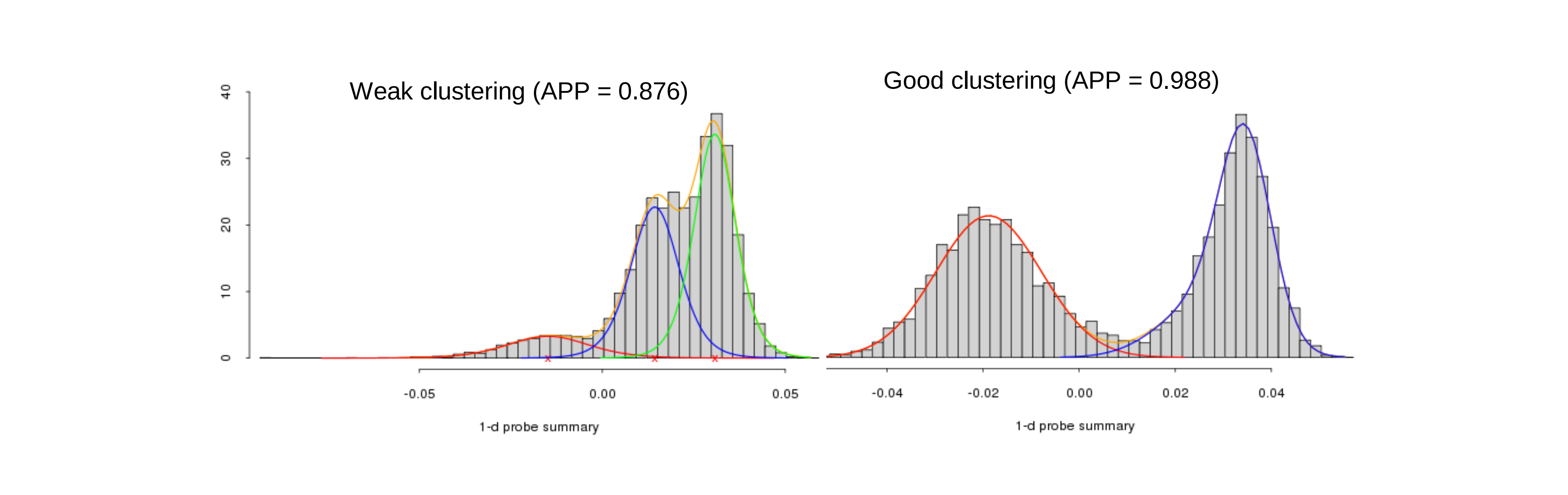

Figure S3: Clustering Quality. The CNVR of the left clustered into 3 classes but with weak separation between classes potentially leading to incorrect copy number assignment reflected by the low APP value. The CNVR on the right has clustered well into 2 distinct classes and will be more likely to give accurate copy number assignment for most samples.

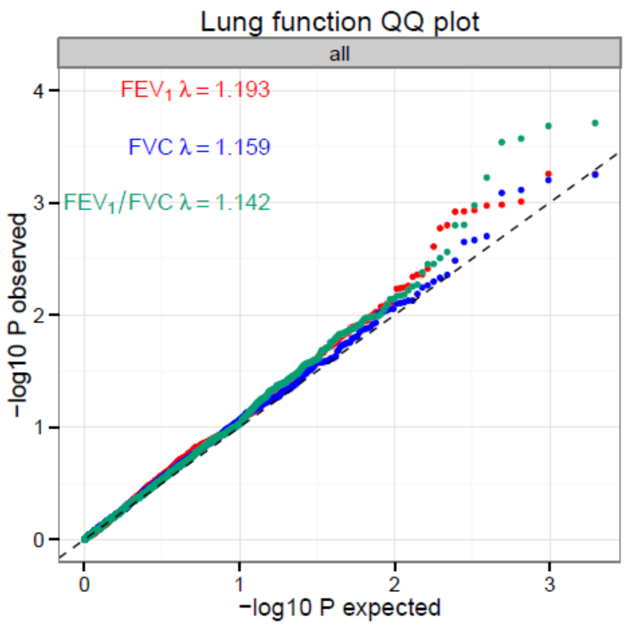

Figure S4: QQ plot for lung function meta-analysis

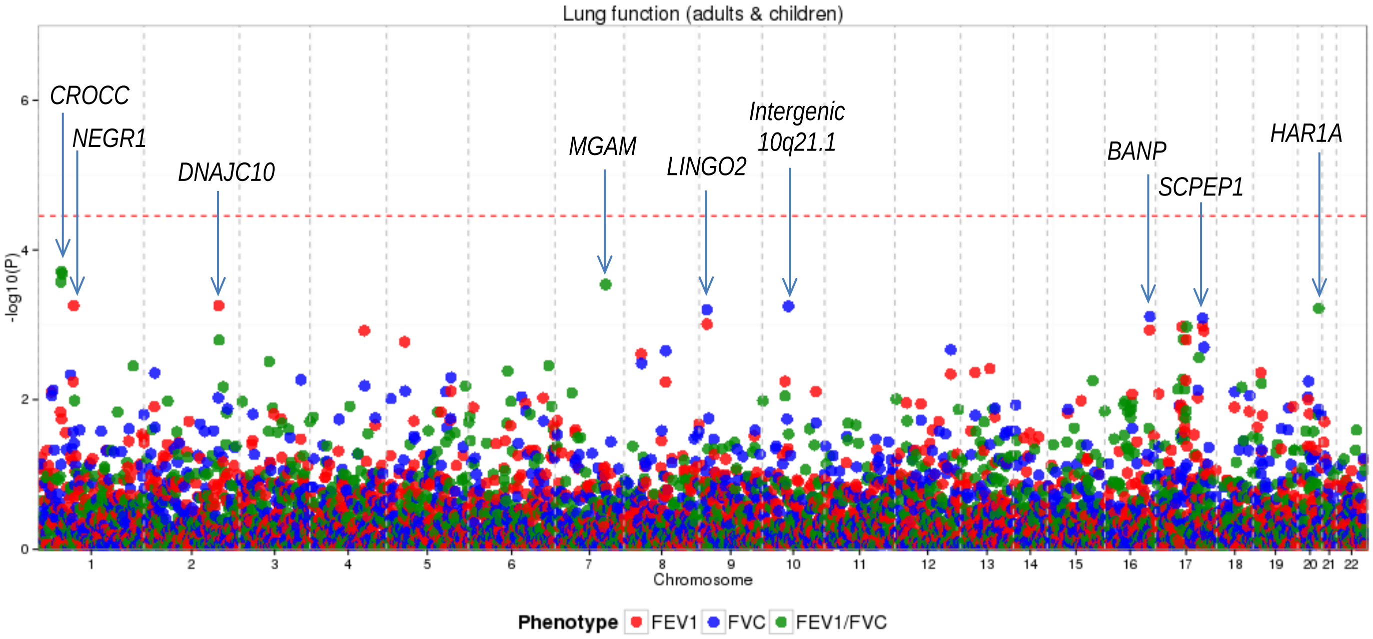

Figure S5: Manhattan plot for lung function meta-analysis across 4 adult and child cohorts. Genome-wide significance for 1447 independent tests P = 3.5×10^{-5} shown as red line.

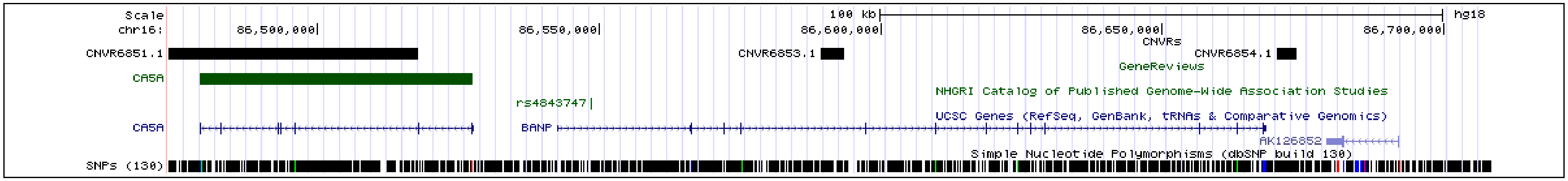

Figure S6: Location of CNVR6854.1 downstream of *BANP*

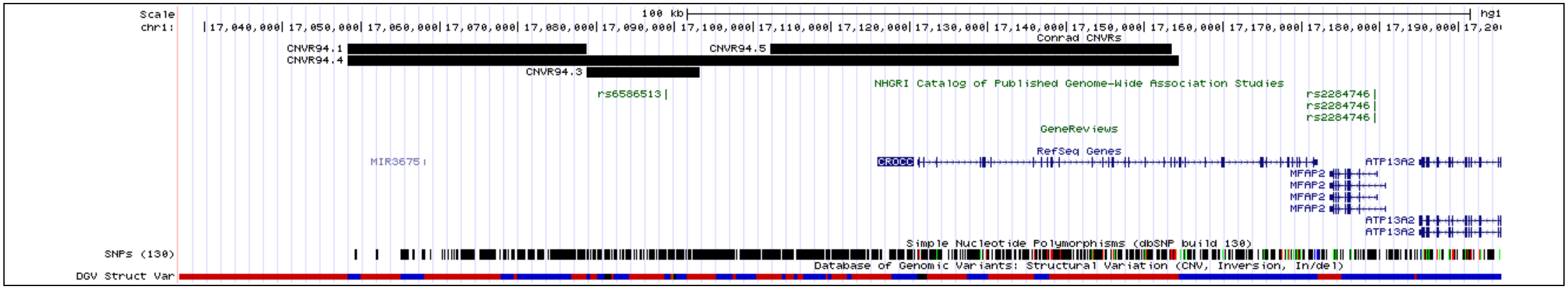

Figure S7: Location of CNVRs relative to *CROCC*
The *CROCC* region has nested CNVRs both overlapping the *CROCC* gene and upstream of it.

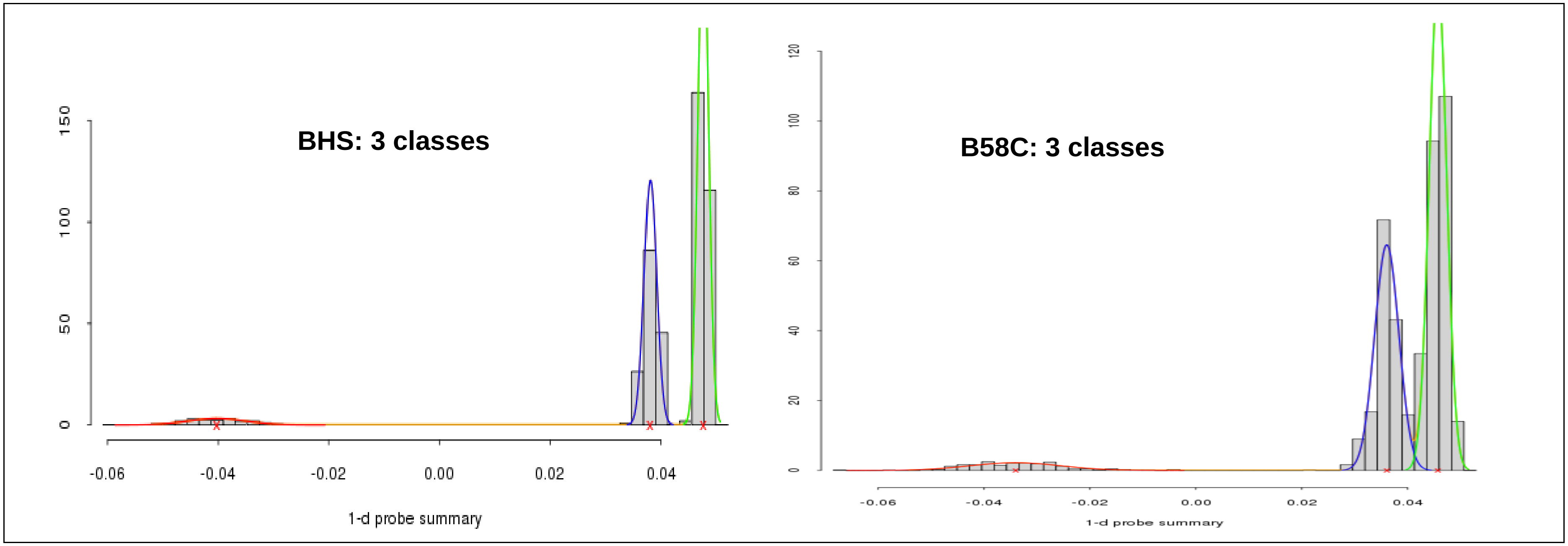

Figure S8: CNVR6854.1 at 16q24.2 (downstream *BANP*). Good resolution of a 3 class CNVR.

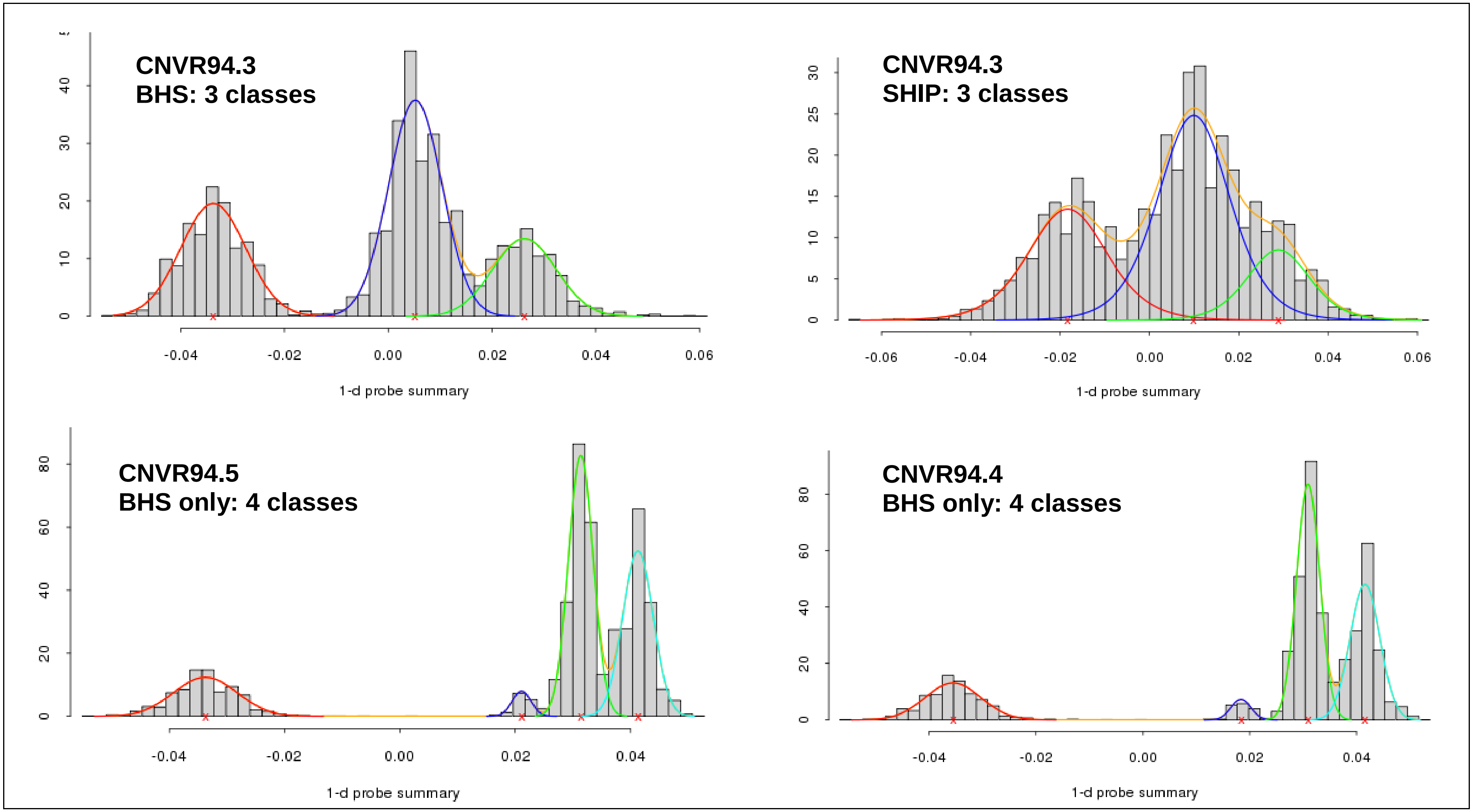

Figure S9: Three CNVRs at 1p36.13 (*CROCC*). CNVR94.3 was well clustered in BHS (top left) but weakly clustered in SHIP (top right). CNVR94.5 (bottom left) and CNVR94.4 (bottom right) were well clustered in BHS but were not able to be clustered in any other cohorts.

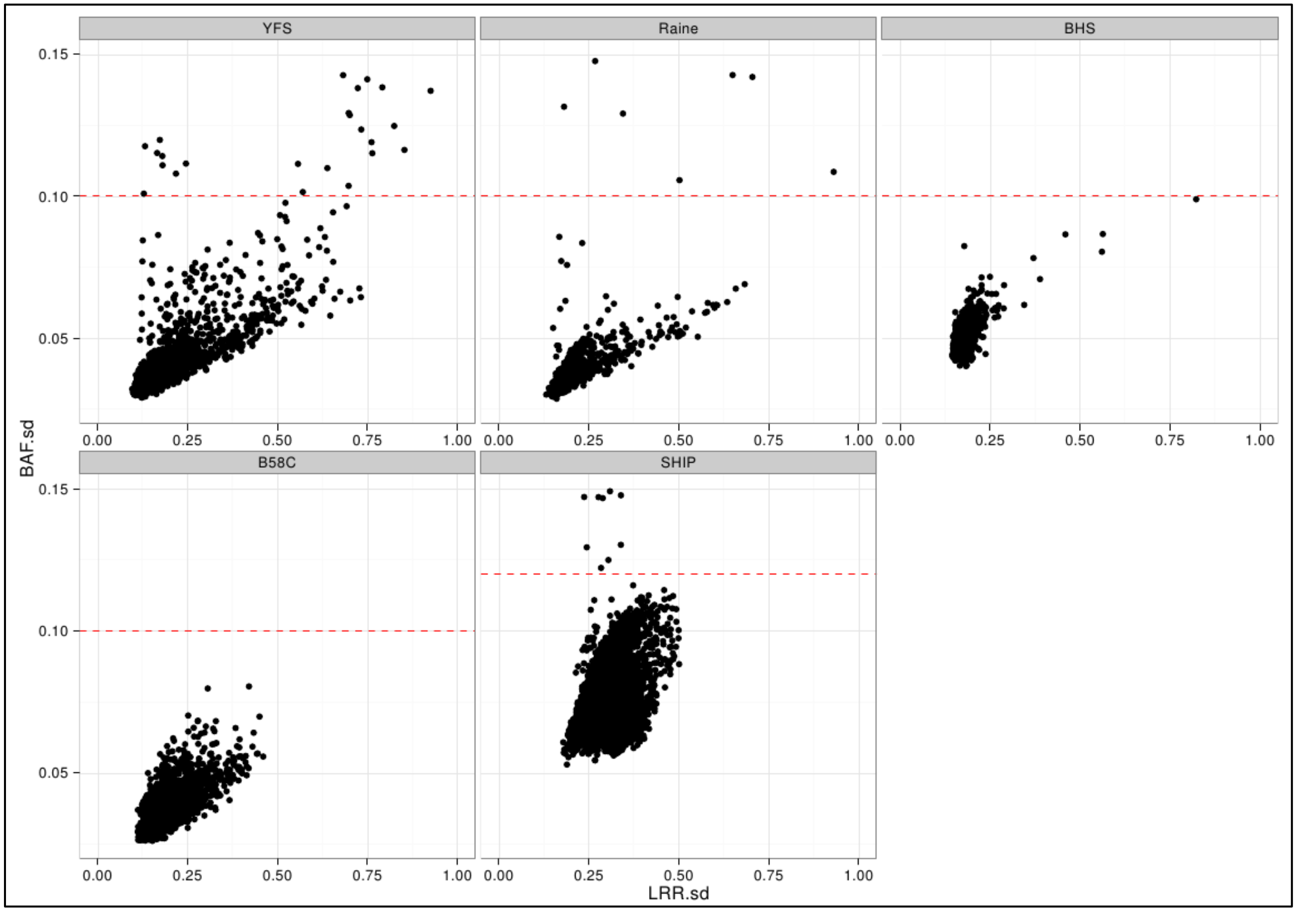

Figure S10: Standard deviation of Log R Ratio (LRR.sd) and B Allele Frequency (BAF.sd) across probes (one point per sample) to compare intensity noise between cohorts. The red dotted line shows the BAF.sd thresholdused for exclusion. For samples genotyped on Illumina platforms we used a BAF sd threshold of 0.1 and for Affymetrix 0.12 (SHIP only).

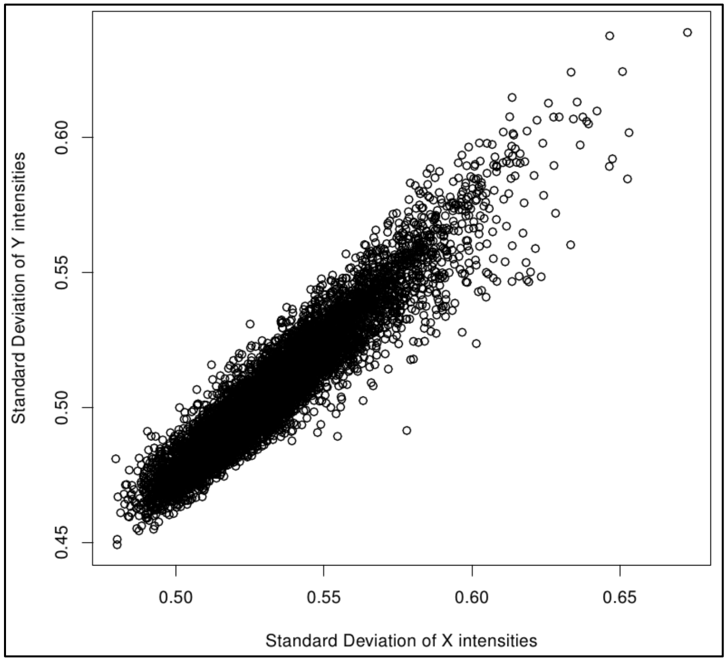

Figure S11: Standard deviation of X and Y intensities for ALSPAC
